# The Association Between Eating Quickly and Excessive Gestational Weight Gain

**DOI:** 10.1089/whr.2023.0003

**Published:** 2023-06-02

**Authors:** Eri Abe, Minatsu Kobayashi, Reiko Horikawa, Naho Morisaki, Hisako Tanaka, Haruhiko Sago, Kohei Ogawa, Takeo Fujiwara

**Affiliations:** ^1^ Graduate School of Studies in Human Culture, Otsuma Women's University, Tokyo, Japan.; ^2^ Department of Social Medicine, National Research Institute for Child Health and Development, Tokyo, Japan.; ^3^ Department of Food Science, Otsuma Women's University, Tokyo, Japan.; ^4^ Division of Endocrinology and Metabolism, National Center for Child Health and Development, Tokyo, Japan.; ^5^ Department of Global Health Promotion, Tokyo Medical and Dental University, Tokyo, Japan.

**Keywords:** dietary behavior, eating rate, pregnant women, gestational weight gain

## Abstract

**Objectives:**

Maintaining an appropriate gestational weight gain (GWG) is essential for a safe pregnancy and delivery. This study aimed to determine the association between the habit of eating quickly and the risk of excessive GWG.

**Methods:**

We administered a questionnaire on eating habits to 1246 pregnant Japanese women in their second to third trimesters. We categorized the participants into three groups according to their answers to the question “Do you eat quickly?” Group 1, “always” or “usually”; Group 2, “sometimes”; and Group 3, “rarely” or “never.” We assessed GWG according to the “The Optimal Weight Gain Chart” (Ministry of Health, Labor and Welfare, Japan), and those who exceeded the criteria were considered “excessive.” Logistic regression analysis was performed with the risk of excess GWG as the dependent variable and quick food intake as the independent variable, to obtain relevant odds ratios (ORs) and 95% confidence intervals (CIs). Model 1 was unadjusted, and Model 2 was adjusted for age, prepregnancy body mass index, energy intake, mother's educational attainment, household income, exercise habits, and childbearing experience.

**Results:**

The OR (95% CI) for Groups 2 and 3 in Model 1, compared with Group 1, was 0.80 (0.62–1.05) and 0.61 (0.43–0.88), respectively (*p* for trend = 0.047). In Model 2, the OR (95% CI) for Groups 2 and 3 were 0.73 (0.55–0.96) and 0.59 (0.40–0.86), respectively (*p* for trend = 0.003).

**Conclusion:**

These results suggest that quick food ingestion increases the risk of excessive GWG.

## Background

Maintaining appropriate gestational weight gain (GWG) is essential for safe pregnancy and delivery. Excessive GWG increases the risk of gestational diabetes mellitus (GDM), preeclampsia, macrosomia, and need for cesarean delivery.^1,2^ GDM is a pregnancy complication that pregnant Asian women should be particularly aware of. Compared with other racial groups, Asian women have a much higher risk of developing GDM^3–5^ and subsequent type 2 diabetes.^6^ Excess GWG has also been reported to have an impact after delivery, with an increased risk of obesity lasting 15 years postpartum.^7^ Therefore, GWG is critical for safe pregnancy and delivery, as well as the general future health of the mother.

During pregnancy, energy and nutrient requirements increase owing to fetal growth and physiological changes in the mother. The Dietary Reference Intakes for Japanese 2020 set additional amounts of energy, protein, vitamin A, vitamin B_1_, vitamin B_2_, vitamin B_6_, vitamin B_12_, folic acid, vitamin C, magnesium, iron, zinc, copper, and iodine for women during pregnancy.^8^ The Ministry of Health, Labor, and Welfare of Japan has issued the “Dietary Guidelines for Pre-pregnant, Pregnant and Lactating Women in Japan, 2020 ∼ Get health conscious before your pregnancy ∼” to ensure appropriate dietary habits during pregnancy.^9^

The guidelines provide dietary recommendations such as “Achieve your adequate energy intakes through staple foods,” “Side dishes help you take enough amounts of vitamins and minerals,” and “Arrange main dishes so that you can take enough protein.” Furthermore, the “Food Guide for Pregnant and Lactating Women in Japan” indicates the standard amount (No. of servings) of staple foods, main dishes, and side dishes consumed per day during pregnancy.^10^ However, as described earlier, in Japan, although there is an awareness of the nutrients and dietary content that should be consumed during pregnancy, there is little mention of the rate of food intake.

A meta-analysis of the association between obesity and eating speed reported that those who ingested food quickly were more likely to have obesity.^11^ It has been suggested that excess energy intake caused by quick food intake increases the risk of obesity.^12^ Self-reported quick eating rates have also been associated with an increased risk of metabolic diseases. Self-reported quick eating rates have been reported to be associated with increased insulin resistance,^13^ metabolic syndrome,^14,15^ nonalcoholic fatty liver disease,^16,17^ and abnormal blood lipid metabolism.^18^

In the Obesity Treatment Guidelines 2016 (Japan),^19^ the eating behavior of chewing food slowly and thoroughly is indicated as behavioral therapy for obesity control. Therefore, eating rate is a vital eating behavior, even during the heightened energy requirements of pregnancy when proper weight control is required in a short period of ∼10 months. However, the relationship between the eating rate and GWG has not yet been reported. This study investigated the association between eating quickly and the risk of excess pregnancy weight gain among pregnant Japanese women.

## Methods

### Study design

This study used data from a prospective cohort study conducted at the National Center for Child Health and Development (Setagaya-Ku, Tokyo, Japan) between May 2010 and November 2013. Participants were randomly recruited at the obstetric outpatient clinic between 8 and 15 weeks of gestation at the first visit. A baseline survey was administered at the time of recruitment, followed by a lifestyle and dietary survey during the second survey from the second to the third trimester (26–35 weeks gestation).

### Baseline survey

Information on age (years), height (cm), prepregnancy weight (kg), annual household income (<4 million yen, 4–8 million yen, 8–10 million yen, >10 million yen, and missing), and mother's educational level (high school, junior college, university or graduate school, and missing) was obtained at the time of the baseline survey using a self-administered questionnaire. Prepregnancy body mass index (BMI) was calculated using the following formula: prepregnancy weight (kg)/height (m)^2^.

### Lifestyle survey

The lifestyle survey included a question regarding exercise habits: “Do you exercise for at least 15 minutes a week?” with “yes” and “no” response options. The survey also included a question regarding eating speed: “Do you eat quickly?” with five options: “always,” “usually,” “sometimes,” “rarely,” and “never.”

### Dietary survey

We conducted a dietary survey using the semiquantitative Food Frequency Questionnaire (FFQ), which has reported external validity in Japanese pregnant women, to ascertain habitual energy and nutrient intake for the 2 months preceding survey administration.^20,21^ Spearman's correlation coefficients of nutrients ranged from 0.098 (sodium) to 0.401 (vitamin C), and all of the 36 nutrients were statistically significant. In 27 food groups, the correlation coefficients ranged from −0.015 (alcohol) to 0.572 (yogurt), and 81% were statistically significant.

Participants responded to the frequency of intake of 165 different foods and the amount consumed per serving for the preceding 2 months. There were nine options for the frequency of intake of each food: (1) less than once a month, (2) 1–3 times per month, (3) 1–2 times per week, (4) 3–4 times per week, (5) 5–6 times per week, (6) once per day, (7) 2–3 times per day, (8) 4–6 times per day, and (9) 7 or more times per day. The amount of food consumed per serving for each food type was divided into three categories: “less (less than half),” “same,” and “more (more than 1.5 times),” compared with the reference amount indicated for each food. Based on the Japanese Standard Tables of Food Composition (2015 edition),^22^ the respondents' daily intakes of energy, protein, fat, and carbohydrate were calculated using the weighted average food composition table prepared for this study. Nutrient intake was also energy-adjusted using the density method, to eliminate the influence of energy intake.

### Evaluation of GWG

The GWG of each participant was calculated by subtracting their self-reported prepregnancy weight from their weight at delivery. We used weight-at-delivery data from the medical records of the delivery center. In addition, we assessed GWG according to the “Optimal Weight Gain Chart During Pregnancy”^23^ provided by the Ministry of Health, Labor and Welfare (Japan) at the time the study was conducted (2010 − 2013). According to this chart, the optimal weight gain during pregnancy was 9 − 12 kg for pregnant women with a prepregnancy BMI ≤18.5, 7 − 12 kg for those with a BMI of 18.5 − 25.0, and <5 kg for those with a BMI ≥25.0.

GWG above the standard values shown in the optimal weight gain chart was rated as “excessive,” weight gain within the standard range was rated as “adequate,” and weight gain below the standard was rated as “insufficient.”

### Statistical analysis

A total of 1563 women agreed to participate in the study, and 1366 completed the FFQ between 26 and 35 weeks of gestation. After excluding multiple pregnancies (*n* = 94), those with incomplete prepregnancy weights (*n* = 14), and those with extremely low and high energy intake (1% above and below) (*n* = 12), 1246 (79.7%) women were eligible for analysis.

We classified the participants according to their answers to the question “Do you eat quickly?” with those who answered “always” or “usually” in Group 1, those who answered “sometimes” in Group 2, and those who answered “rarely” or “never” in Group 3.

Because the age and weight gain at conception variables had non-normal distributions, the Kruskal–Wallis nonparametric test was used to compare the three groups. The chi square test was used for the categorical variables of prepregnancy body size, assessment of weight gain during pregnancy, education level, household income, previous childbearing experience, and exercise habits. Since energy and nutrient intakes were non-normally distributions, the Steel–Dwass test was used for nonparametric multiple comparisons.

To determine the association between quick food intake and the risk of excess GWG, logistic regression analysis was performed, with the risk of excess GWG as the dependent variable and the habit of eating quickly as the independent variable.

The odds ratios (ORs) and 95% confidence intervals (CIs) for the risk of excess GWG in Groups 2 and 3 were determined using Group 1, which had the highest frequency of eating quickly, as the reference group.

Model 1 was unadjusted, and Model 2 was adjusted for age (years), prepregnancy BMI (underweight [<18.5], normal [18.5–24.9], and overweight [≥25]), energy intake during the second half of pregnancy (kcal), mother's education level (high school, junior college, university/graduate school, and missing), annual household income (<4 million yen, 4–8 million yen, 8–10 million yen, >10 million yen, and no entry), exercise habits of >15 minutes per week (yes or no), and previous childbearing experience (yes or no). All statistical analyses were performed using JMP13 (SAS Institute, Cary, NC) with a significance level of *p* < 0.05.

## Results

Group 1 had 178 (14.3%), Group 2 had 506 (40.6%), and Group 3 had 562 (45.1%) participants. The mean age (standard deviation [SD]) was 35.8 (4.3) years, and the mean BMI (SD) was 20.4 (2.6).

Table 1 shows the characteristics of the participants in each of the three groups, according to their habits of eating quickly. Age, prepregnancy BMI, previous childbearing experience, educational level, and annual household income were significantly different between the groups.

**Table 1.** Characteristics of Pregnant Women According to the Habit of Eating Quickly (*n* = 1246)

	Group 1	Group 2	Group 3	
	*n* = 178	*n* = 506	*n* = 562	
	Mean (SD)	Mean (SD)	Mean (SD)	*p* ^a^
Age (years)	36.0 (4.1)	36.3 (4.2)	35.5 (4.5)	0.015
GWG (kg)	10.2 (4.1)	9.7 (3.6)	9.8 (3.4)	0.517

	*n* (%)	*n* (%)	*n* (%)	
Prepregnant BMI (kg/m^2^)				
Underweight (<18.5)	28 (15.7)	100 (19.8)	130 (23.1)	0.038
Normal (18.5–24.9)	135 (75.8)	385 (76.1)	411 (73.1)	
Overweight (≥25)	15 (8.4)	21 (4.2)	21 (3.7)	
Evaluate of GWG (kg)				
Insufficient	40 (22.5)	118 (23.3)	131 (23.3)	0.077
Appropriate	72 (40.4)	231 (45.7)	282 (50.2)	
Excess	66 (37.1)	157 (31.0)	149 (26.5)	
Previous childbearing experience				
Yes	107 (60.1)	217 (42.9)	147 (26.2)	<0.0001
No	71 (39.9)	289 (57.1)	415 (73.8)	
Mother's educational attainment				
High school	21 (11.8)	27 (5.3)	41 (7.3)	0.030
Junior college	57 (32.0)	131 (25.9)	166 (29.5)	
University, graduate school	91 (51.1)	323 (63.8)	325 (57.8)	
Missing	9 (5.1)	25 (4.9)	30 (5.3)	
Annual household income (JPY)				
<4 million	17 (9.6)	22 (4.3)	52 (9.3)	0.002
4 million to <8 million	54 (30.3)	147 (29.1)	205 (36.5)	
8 million to <10 million	26 (14.6)	101 (20.0)	92 (16.4)	
≥10 million	65 (36.5)	185 (36.6)	159 (28.3)	
Missing	16 (9.0)	51 (10.1)	54 (9.6)	
Physical activity				
≥15 minutes/week	37 (20.8)	130 (25.7)	145 (25.8)	0.469
<15 minutes/week	127 (71.3)	335 (66.2)	362 (64.4)	
Missing	14 (7.9)	41 (8.1)	55 (9.8)	

The three groups are based on responses to the question “Do you eat quickly?”: Group 1, “always” or “usually”; Group 2, “sometimes”; and Group 3, “rarely” or “never.”

^a^ Chi square test for categorical variables, Kruskal−Wallis test for continuous variables.

BMU, body mass index; GWG, gestational weight gain; SD, standard deviation.

Group 1, which had the highest frequency of eating quickly, tended to include older participants, had obesity before pregnancy, and had an annual household income of >10 million yen. Group 1 also had fewer participants with previous childbearing experience and university-level education than Group 3, which had the lowest frequency of eating quickly. Sixty-six participants (37.1%) had excessive GWG in Group 1, 157 (31.0%) in Group 2, and 149 (26.5%) in Group 3.

Table 2 shows nutrient intakes by the three groups, according to their eating habits. Groups 1 and 2 had significantly higher energy, protein, and carbohydrate intakes than Group 3. However, after adjusting for energy intake using the density method, there were no significant differences in nutrient intake between the three groups.

**Table 2.** Nutrient Intakes by Three Groups According to the Habit of Eating Quickly

	Group 1	Group 2	Group 3	*p* ^a^		
	*n* = 178	*n* = 506	*n* = 562			
	Mean (SD)	Mean (SD)	Mean (SD)	1–2	1–3	2–3
Crude						
Energy (kcal)	1796 (573)	1716 (581)	1634 (590)	0.189	0.001	0.007
Protein (g)	62.0 (23.7)	60.9 (26.0)	56.4 (23.0)	0.625	0.011	0.015
Fat (g)	59.4 (28.7)	59.3 (30.1)	56.6 (30.5)	0.996	0.288	0.118
Carbohydrate (g)	248.6 (77.5)	229.8 (73.3)	220.7 (74.5)	0.006	<0.0001	0.020
Energy density value						
Protein (E%)^b^	13.6 (2.0)	14.0 (2.2)	13.7 (2.1)	0.196	0.845	0.236
Fat (E%)^b^	29.1 (7.9)	30.1 (8.1)	30.1 (7.9)	0.365	0.289	0.995
Carbohydrate (E%)^b^	56.3 (9.5)	54.8 (9.9)	55.3 (9.5)	0.221	0.431	0.772

The three groups are based on responses to the question “Do you eat quickly?” Group 1, “always” or “usually”; Group 2, “sometimes”; and Group 3, “rarely” or “never.”

^a^ Steel−Dwass test.

^b^ Energy%.

The risk of excessive GWG for the three groups according to their self-reported habits of eating quickly is shown in Table 3. The ORs in the unadjusted model for Group 2 and Group 3 were 0.80 (95% CI: 0.62–1.05) and 0.61 (95% CI: 0.4 − 0.88), respectively, compared with Group 1. In Model 2, adjusted for age prepregnancy BMI, energy intake, annual household income, education level, exercise habits, and previous childbearing experience, the ORs for Groups 2 and 3 were 0.73 (95% CI: 0.55–0.96) and 0.59 (95% CI: 0.40–0.86), respectively.

**Table 3.** Odds Ratios and 95% Confidence Intervals for the Risk of Excess Gestational Weight Gain

		OR	95% CI	*p* ^a^	*p*-for trend
Model 1	Group 1	Ref.			
	Group 2	0.80	0.62–1.05	0.104	0.047
	Group 3	0.61	0.43–0.88	0.007	
Model 2	Group 1	Ref.			
	Group 2	0.73	0.55–0.96	0.025	0.003
	Group 3	0.59	0.40–0.86	0.006	

The three groups based on responses to the question “Do you eat quickly?”: Group 1, “always” or “usually”; Group 2, “sometimes”; and Group 3, “rarely” or “never.”

^a^ Logistic regression analysis.

Model 1: Unadjusted.

Model 2: Age, prepregnancy BMI, energy intake, annual household income, mother's educational levels, exercise habit, and childbearing experience were adjusted.

CI, confidence interval; OR, odds ratio.

## Discussion

In this study, pregnant women who self-reported eating quickly were found to be at a higher risk of excess weight gain during pregnancy. The association between frequency of eating quickly and excess GWG remained significant after adjusting for age, prepregnancy BMI, energy intake in the second trimester of pregnancy, education level, annual household income, exercise habits, and previous childbearing experience.

The group with the highest frequency of quick eating had a significantly higher energy intake. A recent meta-analysis that examined the relationship between eating speed and energy intake also reported that eating quickly was associated with increased energy intake.^12^

The association between eating quickly and increased energy intake may be due to increased stomach distention, the release of cholecystokinin, glucagon-like peptide-I, and peptide YY, or elevated blood glucose levels. All of these factors may result in a higher energy intake before the hypothalamic satiety center is stimulated.^24,25^

It has been reported that the same amount of the same food, consumed for a longer intake period, results in increased secretion of glucagon-like peptide-I and peptide YY, and a greater sense of satiety.^26^ Decreased chewing due to eating quickly may also influence increased energy intake. Chewing activates histamine neurons and suppresses appetite through the satiety center.^27^ Chewing more than usual has been reported to help people feel satisfied with less food intake.^28,29^ Therefore, eating quickly may decrease the amount of chewing, in turn resulting in less satiety, increased energy intake, and excessive GWG.

People with high energy intake have been reported to consume diets with high energy density (kcal/minutes).^30^ In our study, participants in Groups 2 and 3, who self-reported eating quickly, may have consumed foods with higher energy density than those in Group 1. For example, confectioneries and juices are classified as high energy density (kcal/minutes) foods, whereas vegetables and teas are classified as low energy density foods.^30^ However, there were no significant differences observed between the three groups in terms of consumption of confectioneries, juices, vegetables, or teas in this study (data not shown).

Carbohydrates contributed significantly to the increase in energy intake observed due to self-reported quick food intake among the participants in this study. A comparison of daily intakes of staple foods such as rice, bread, and noodles among the three groups revealed significant differences only with regard to rice intake. The mean (SD) rice intake (g) for Groups 1–3 were 272 (144), 247 (118), and 231 (108), respectively, with Group 1 having a significantly higher consumption than Group 2 (*p* = 0.005). Since rice is a relatively soft food that requires less chewing, the consumption of rice (a staple food for Japanese people) may have increased among pregnant women who self-reported eating quickly. Therefore, educating participants who would benefit from spending more time on meals to be aware of the appropriate amount of staple food to consume may help prevent overeating.

By contrast, the association between the frequency of eating quickly and the risk of excess GWG remained significant after adjusting for energy intake in this study. Studies of middle-aged Japanese adults,^31^ 18-year-old Japanese women,^32^ and Korean adults^18^ have also reported associations between eating rate and obesity, even after adjusting for energy intake. This suggests that the association between eating rate and excess GWG may have causes other than simply excessive energy intake. Intervention studies have also reported that less chewing and shorter meal duration reduce postprandial visceral blood flow and decrease diet-induced thermogenesis.^33^ In addition, animal experiments have reported that a decrease in chewing, due to eating quickly, causes the inactivation of neuroleptic histamine and suppresses lipolysis in visceral adipose tissue.^34^

In this study, the multiparas women were more likely to self-report eating quickly. For example, in Group 3, the group with the lowest frequency of eating quickly, ∼30% were multipara, whereas in Group 1, the group with the highest frequency of eating quickly, ∼60% of participants were multipara. For second and subsequent pregnancies, having to care for another child may not allow mothers enough time for slower meals. When Model 2 was analyzed, stratified by primipara and multipara to eliminate the effect of previous childbirth experience, the ORs were 0.79 (95% CI: 0.60–0.98) for Group 2 and 0.59 (95% CI: 0.42–0.87) for Group 3 among primipara, and 0.69 (95% CI: 0.42–0.97) for Group 2 and 0.54 (95% CI: 0.31–0.95) for Group 3 among multipara, all of which were significantly different (*p* = 0.044, 0.034).

The risk of excessive GWG due to eating quickly was not significantly different between the primipara and multipara. Obtaining family support to ensure adequate meal lengths and using housekeeping services may help prevent eating quickly, not only for multiparas, but also for primiparas. However, this study did not examine whether the multiparas subjects changed their eating speeds after their previous births. Further research is needed to determine whether the burden of caring for children affects eating speed.

One major strength of this study is that the survey was conducted on an ongoing basis at the hospital where the participants gave birth. Dietary surveys and questionnaires were administered during pregnancy, and the participants' weights were measured immediately before delivery.

Nevertheless, this study also has several limitations. First, the participants' prepregnancy weights were self-reported. However, a study of Japanese adults (1148 women and 4253 men) reported a very high correlation (*r* = 0.959) between self-reported and measured weights in women.^35^ Thus, self-reported weight likely has significant validity. The assessment of quick food intake habits was self-reported rather than objective. However, self-reported eating speeds have been reported to be consistent with those reported by friends, in other studies.^32^

In addition, other validation studies have also shown correlations between self-reported eating time and laboratory-measured and recalled eating time^36^; therefore, there is likely significant reliability in self-reported eating rates. Furthermore, underreporting of energy intake is inevitable in self-reported dietary surveys. It has been reported to occur in dietary surveys using the FFQ,^37^ and the effect of underreporting can be assumed in this self-administered FFQ survey as well. A survey of pregnant Japanese women reported that the frequency of underreporting was higher among those who had gained more weight during pregnancy in the second trimester,^38^ and it is possible that the effect of underreporting of energy intake could not be eliminated in this study.

Regarding physical activity, this study investigated whether the women had exercise habits of at least 15 minutes per week, but did not ascertain the intensity of exercise, the time required for exercise, or the energy consumed during daily activities. Therefore, it is possible that differences in energy consumption may affect the GWG. Furthermore, it should be noted that this study was conducted in the urban area of Setagaya-Ku, Tokyo, and the participants were older and had a higher income.^39^ Higher socioeconomic status may have influenced the GWG due to healthier habits, since the risk of inappropriate eating habits and physical inactivity is lower with higher socioeconomic status.^40^

Future studies should determine whether similar trends are observed in pregnant populations with other attributes. Since many pregnant women in this study who self-reported eating quickly were multiparous, it would be important to investigate whether eating speed changes before and after the birth of the first child in the future. In addition, investigating how diet differs between pregnant women who eat quickly and those who do not, including energy density (kcal/minutes), will lead to specific intervention strategies for pregnant women.

## Conclusion

This study showed that eating quickly is an eating behavior that increases energy intake and is a risk factor for excess GWG. In addition to a desirable nutritional balance and dietary content, education about eating rates is also expected to aid weight management during pregnancy.

## Data Availability

The data supporting the findings of this study are available upon request from the corresponding author. The data were not publicly available because of ethical restrictions.

## Abbreviations Used

BMI: body mass index
CI: confidence interval
FFQ: Food Frequency Questionnaire
GWG: gestational weight gain
OR: odds ratio
SD: standard deviation
